# Computer-based facial recognition as an assisting diagnostic tool to identify children with Noonan syndrome

**DOI:** 10.1186/s12887-024-04827-7

**Published:** 2024-05-24

**Authors:** Yulu Huang, Haomiao Sun, Qinchang Chen, Junjun Shen, Jin Han, Shiguang Shan, Shushui Wang

**Affiliations:** 1 Department of Pediatric Cardiology, Guangdong Cardiovascular Institute, Guangdong Provincial People’s Hospital, Guangdong Academy of Medical Sciences, No. 106, Zhongshan 2nd Road, Yuexiu District, Guangzhou, China; 2 Department of Pediatric Cardiology, Guangdong Provincial People’s Hospital (Guangdong Academy of Medical Sciences), Southern Medical University, No. 106, Zhongshan 2nd Road, Yuexiu District, Guangzhou, China; 3 Key Laboratory of Intelligent Information Processing, Institute of Computing Technology, Chinese Academy of Sciences, No. 6 South Science Academy Road, Haidian District, Beijing, China; 4 University of Chinese Academy of Sciences, No. 80 Zhongguancun Road East, Haidian District, Beijing, China; 5 Prenatal diagnosis center, Guangzhou Women and Children’s Medical Center, Guangzhou Medical University, No. 9 Jinsui Road, Tianhe District, Guangzhou, China

**Keywords:** Noonan syndrome, Genetic syndrome, Convolution neural network, Facial recognition, Batch normalization

## Abstract

**Background:**

Noonan syndrome (NS) is a rare genetic disease, and patients who suffer from it exhibit a facial morphology that is characterized by a high forehead, hypertelorism, ptosis, inner epicanthal folds, down-slanting palpebral fissures, a highly arched palate, a round nasal tip, and posteriorly rotated ears. Facial analysis technology has recently been applied to identify many genetic syndromes (GSs). However, few studies have investigated the identification of NS based on the facial features of the subjects.

**Objectives:**

This study develops advanced models to enhance the accuracy of diagnosis of NS.

**Methods:**

A total of 1,892 people were enrolled in this study, including 233 patients with NS, 863 patients with other GSs, and 796 healthy children. We took one to 10 frontal photos of each subject to build a dataset, and then applied the multi-task convolutional neural network (MTCNN) for data pre-processing to generate standardized outputs with five crucial facial landmarks. The ImageNet dataset was used to pre-train the network so that it could capture generalizable features and minimize data wastage. We subsequently constructed seven models for facial identification based on the VGG16, VGG19, VGG16-BN, VGG19-BN, ResNet50, MobileNet-V2, and squeeze-and-excitation network (SENet) architectures. The identification performance of seven models was evaluated and compared with that of six physicians.

**Results:**

All models exhibited a high accuracy, precision, and specificity in recognizing NS patients. The VGG19-BN model delivered the best overall performance, with an accuracy of 93.76%, precision of 91.40%, specificity of 98.73%, and F1 score of 78.34%. The VGG16-BN model achieved the highest AUC value of 0.9787, while all models based on VGG architectures were superior to the others on the whole. The highest scores of six physicians in terms of accuracy, precision, specificity, and the F1 score were 74.00%, 75.00%, 88.33%, and 61.76%, respectively. The performance of each model of facial recognition was superior to that of the best physician on all metrics.

**Conclusion:**

Models of computer-assisted facial recognition can improve the rate of diagnosis of NS. The models based on VGG19-BN and VGG16-BN can play an important role in diagnosing NS in clinical practice.

**Supplementary Information:**

The online version contains supplementary material available at 10.1186/s12887-024-04827-7.

## Introduction

Patients suffering from most genetic syndromes (GSs) have characteristic craniofacial appearances, and the facial dysmorphia in people with the same GS is usually similar [1]. In 2003, Loos et al. [2] were the first to use a machine learning-based method to classify five syndromes with an accuracy of 76%. Advances in the technologies used for facial analysis have spawned a large number of studies on automatic facial recognition for the identification of GSs [3–5]. With improvements in data storage and computational power in recent years, the convolution neural network (CNN) has emerged as the most important method of facial recognition. Unlike traditional methods of machine learning, the CNN can automatically extract the most discriminative features from the input images by using the convolution operation. In 2020, Qin et al. [6] used the CNN to develop a model for the identification of Down syndrome (DS) from the facial features of subjects. It achieved an accuracy of 95.87% and a specificity of 97.40% in distinguishing subjects with DS from healthy subjects. In 2021, Pan et al. [7] established an automatic system based on the CNN to identify patients with Turner syndrome (TS) based on their facial features. It obtained an accuracy of 96.9% and a specificity of 97%. These studies show that facial recognition can be used for the identification of a variety of GSs.

Noonan syndrome (NS) (OMIM: 163,950), first reported by Noonan and Ehmke [8], is one of the most common types of GSs. NS is a genetically heterogeneous disorder, with an estimated prevalence of one in 1,000–2,500, that is caused by germline mutations in the 12 critical genes of the highly conserved Ras/mitogen-activated protein kinases (MAPK) pathway [9]. It is characterized by distinctive facial features, a short stature, congenital heart defect, and developmental delays of varying degrees. Researchers have explored facial recognition based on traditional machine learning for the identification of NS [3, 10–12]. However, few studies have considered the identification of NS based on facial recognition by using the CNN. In past work, our research team developed a model of facial recognition for identifying NS by using the CNN that achieved an accuracy of 81% in distinguishing between NS patients and patients suffering from other GSs^13^. The accuracy of this model needs to be further improved.

## Patients and methods

### Patients and dataset

We constructed a dataset consisting of 3,948 frontal facial images of 233 NS patients, 863 patients suffering from other GSs, and 796 healthy children.

**Fig. 1:**
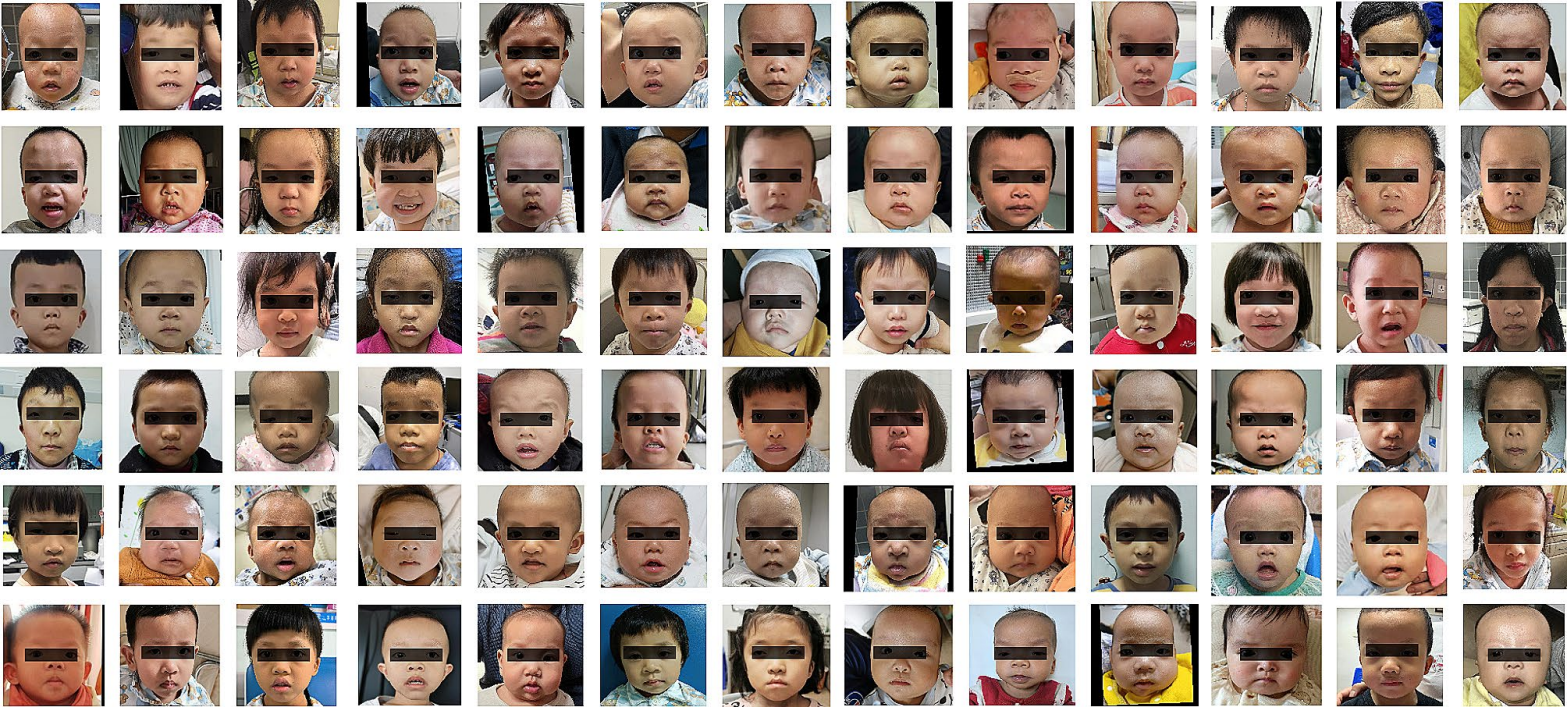
Images of NS patients collected from the Guangdong Provincial Peoples’ Hospital in China (*N* = 78). The black bar has been used to block the patients’ private data

Photographs of 78 NS patients (Figs. 1), 285 patients suffering from other GSs, and 796 healthy children were collected from the Guangdong Provincial Peoples’ Hospital in China from January 2017 to June 2023. We used one to 10 frontal photos of each subject. The diagnoses of NS and the other GSs were confirmed through genetic testing. Nineteen healthy children were subjected to genetic testing to confirm that they did not have any GS, while the other healthy children were evaluated by two pediatric geneticists for the same purpose. Only one patient in our hospital belonged to the Zhuang ethnic group, while all the other GS patients were ethnic Han. We also collected photographs of 37 NS patients from the medical literature [8, 13–19], as well as photographs of 118 NS patients and 578 patients suffering from other GSs from the GestaltMatcher database [20] (GMDB, available at: https://db.gestaltmatcher.org). One frontal photograph of each patient was collected from the literature and the GMDB. All facial images collected from the literature and the GMDB were sufficiently clear for model construction. Information on the ages of the NS patients, data for whom were collected from the literature and the GMDB, was not obtained. We used an online software called Facial Age (https://www.facialage.com/) to estimate their ages. This information is provided in Supplemental File 1.

The training set contained 365 images of 186 NS patients, 2,203 images of 705 patients suffering from other GSs, and 630 images of 630 healthy children. The test set contained 124 images of 47 NS patients, 460 images of 158 patients suffering from other GSs, and 166 images of 166 healthy children. 2,663 images of 863 patients suffering from a total of 78 kinds of other GSs were collected (Table 1). A total of 3,948 images of 1,892 subjects were considered.

**Table 1:** Other genetic syndromes

Other genetic syndromes	Number of cases	Number of images
Williams–Beuren syndrome	173	827
Kabuki syndrome	101	121
Cornelia de Lange Syndrome	54	109
KBG syndrome	52	52
Alagille syndrome	51	134
Wiedemann-Steiner syndrome	50	58
Coffin-Siris syndrome	33	40
Helsmoortel-Van der Aa syndrome	26	75
Mowat-Wilson syndrome	23	23
Down syndrome	21	222
Marfan syndrome	21	152
Fragile X syndrome	15	15
Sotos syndrome	14	14
Myhre syndrome	13	14
Angelman syndrome	13	13
DiGeorge syndrome	12	30
Crouzon syndrome	11	12
Coffin–Lowry syndrome	11	11
Holoprosencephaly	10	10
Rett syndrome	10	10
Costello Syndrome	7	23
Stickler syndrome	7	12
Loeys-Dietz syndrome	6	64
Klippel-Feil syndrome	6	29
Wolf Hirschhorn syndrome	3	17
Other 53 kinds of GSs*	120	576

*There were no cases with Turner syndrome in this study

This study was authorized by the Research Ethics Committee of Guangdong Provincial People’s Hospital (Project No. KY-Z-2020-033-04). Written informed consent was obtained from the guardians of the patients. Permission to use images from the GMDB was also obtained. All facial images collected from the literature and the GMDB were used only for AI-based research.

### Image pre-processing

Image pre-processing consisted of three steps: face detection, data augmentation, and image normalization. Face detection was performed by using the multi-task convolutional neural network (MTCNN), which applies a cascade structure with three multi-task networks: a proposal network (P-Net), a refinement network (R-Net), and an output network (O-Net) [21]. Each image was initially resized to different scales to build an image pyramid. The images were then input to a three-stage cascaded framework. In the first stage, copies of the images were fed to P-Net, which generated candidate bounding boxes containing the faces of the subjects. R-Net, a complex version of the CNN, refined the windows in the second stage to reject a large number of windows that did not feature the faces of the subjects. Finally, O-Net, a more powerful version of the CNN, was applied to process the output of R-Net to extract more features from it, and high-confidence bounding boxes for the face and five landmark points (left eye, right eye, tip of the nose, and left and right corners of the mouth) were generated.

Augmentation technology was used to increase the diversity of the data and expand the sample size. The images were randomly manipulated through rotation, jittering, cropping, and flipping while the facial features of the subjects remained unchanged. The images were normalized to 256 pixels × 256 pixels to match the number of dimensions of the input. The normalized images were then used to construct seven models.

### Architectures

The following CNN architectures were used to construct seven models: VGG16, VGG19, VGG16-BN, VGG19-BN, ResNet50, MobileNet-V2, and SENet.

VGGNet [22] is an architecture that applies small 3 × 3 convolution kernels to capture the receptive field. VGG16 consists of 13 convolutional layers and three fully connected layers. VGG19 adds three convolutional layers to VGG16. Batch normalization (BN) [23] is a mechanism that reduces the variation in the distribution of the inputs to each mini-batch and expedites the training of the neural networks. BN is inserted before every ReLU in the convolutional layers of the VGG16-BN and VGG19-BN models. The mini-batch size was set to 64 in this study. ResNet50 [24] consists of 49 convolutional layers and an average pooling layer. It contains residual blocks in which the inputs can be passed directly to the outputs. The residual blocks avoid the problem of the vanishing gradient and help learn complex features. SENet [25] introduces an architectural unit called the squeeze-and-excitation (SE) block based on ResNet. The SE block performs three sequential operations on the input image: squeezing, excitation, and reweighting. MobileNet [26–28] is a lightweight CNN designed for mobile and embedded devices. MobileNet-V2 introduces a linear bottleneck and an inverted residual structure to avoid losing a large amount of information.

### Construction and testing of CNN models

We used the ImageNet dataset to pre-train the CNN architectures to expedite their convergence by enabling them to capture the general characteristics of the images. The extracted parameters of the facial features were stored in the convolutional and pooling layers, which were frozen to ensure that the parameters of the pre-trained networks could not be adjusted by back-propagation. Pre-training was followed by the initialization of the weights of each CNN model. The last classification layer (softmax) was replaced with a fully connected layer comprising two outputs for binary classification. Each model was then fine-tuned by redesigning and training the fully connected layers on the training set. All models were trained for a maximum of 100 epochs, with a mini-batch size of 64. The training data were randomly shuffled before every epoch. The initial learning rate was set to 0.1, and decayed according to a cosine annealing schedule. At the beginning of each training epoch, we used the initial parameters to perform a forward calculation on all images and recorded the predicted probability of NS for each image. The cross-entropy loss function was subsequently used to calculate the loss between the output labels and the real labels. Following this, the Adam optimizer was used to perform back-propagation and update the model parameters. These steps were repeated until each model finally converged.

The test set was used to evaluate the performance of each model. For each input image, each model provided a probability that predicted whether the corresponding patient suffered from NS. When this probability exceeded 50%, the patient was classified as an NS patient, while the relevant patient was classified as a GS patient or a healthy subject if the probability was below 50%.

### Comparison between models and physicians

Three junior pediatricians (with three to five years of clinical experience) and three senior pediatricians (with more than 15 years of clinical experience) were invited to identify NS patients based on the facial images in the test set. Each image was shown to them without any attendant clinical information. The physicians were then given 10 s to determine whether the individual shown in the photos suffered from NS.

### Evaluation metrics

The results of prediction were divided into four categories: true positive (TP), true negative (TN), false positive (FP), and false negative (FN). Accuracy, precision, specificity, F1 score, the receiver operating characteristic (ROC) curve, and the area under the curve (AUC) were used to evaluate classification performance. The probability of each image in the test set was used to generate the ROC curves, based on which the AUC was computed. These metrics were calculated as follows:

2 $$Accuracy=\frac{TP+TN}{TP+FP+TN+FN}$$

3 $$Precision=\frac{TP}{TP+FP}$$

4 $$Recall=\frac{TP}{TP+FN}$$

5 $$Specificity=\frac{TN}{TN+FP}$$

6 $${F}_{1}=2*\frac{Precision*Recall}{Precision+Recall}$$

**Fig. 2:**
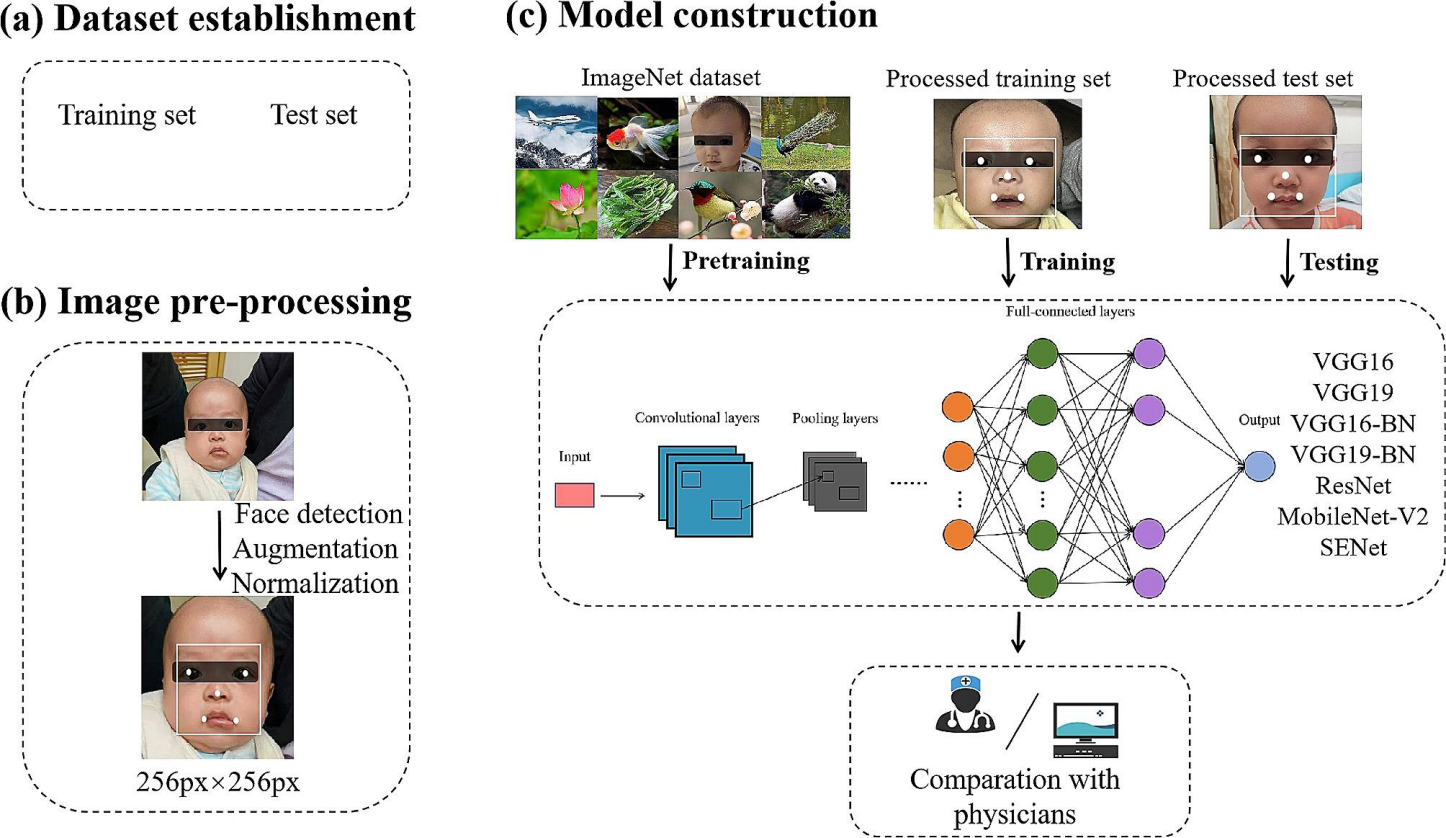
The framework used to construct diagnostic models for NS. **(a)** Dataset establishment. The facial images were split into training and test sets. **(b)** Image pre-processing. Face detection, data augmentation, and image normalization. **(c)** Model construction and evaluation

The framework for constructing the diagnostic models for NS is illustrated in Fig. 2.

## Results

**Fig. 3:**
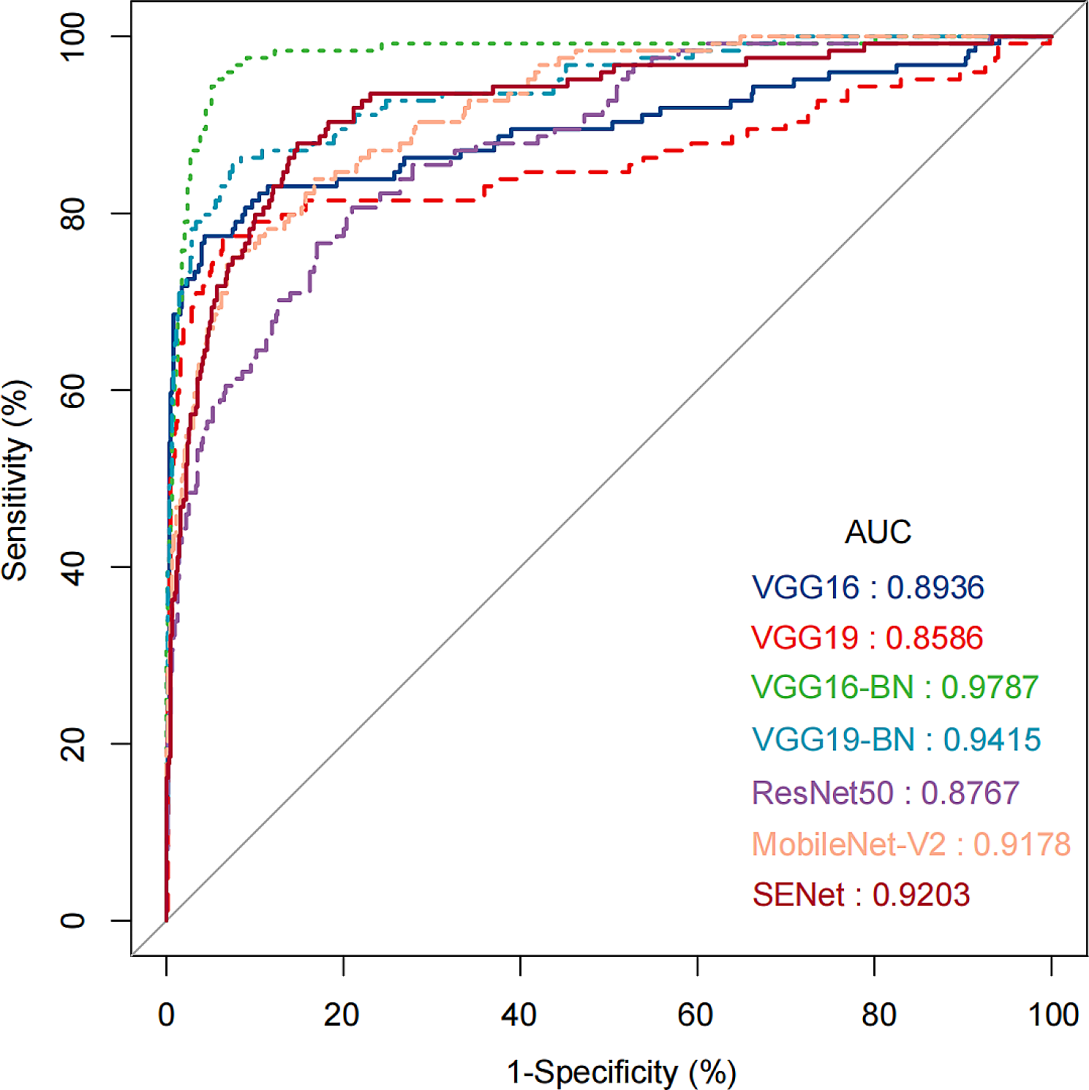
The ROC curves of all CNN models

We constructed seven face recognition-assisted diagnostic models for NS patients by using VGG16, VGG19, VGG16-BN, VGG19-BN, ResNet, MobileNet, and SENet. The accuracy, precision, specificity, and F1 score of each model are presented in Table 2, while their ROC curves are shown in Fig. 3. The VGG19-BN model delivered the best overall performance, with an accuracy of 93.76%, precision of 91.40%, specificity of 98.73%, and F1 score of 78.34%. The VGG16-BN model achieved the highest AUC value of 0.9787. Models based on the VGGNet architectures (VGG16, VGG19, VGG16-BN, and VGG19-BN) outperformed the other models (ResNet50, MobileNet-V2, and SENet) overall.

**Table 2:** Performance of the CNN models

Models	Accuracy %	Precision %	Specificity %	F1%	AUC
VGG16	93.49	88.66	98.25	77.83	0.8936
VGG19	92.83	86.46	97.93	75.45	0.8586
VGG16-BN	92.96	90.80	98.72	74.88	**0.9787**
VGG19-BN	**93.76**	**91.40**	98.73	**78.34**	0.9415
ResNet50	89.11	82.81	98.25	56.38	0.8767
MobileNet-V2	89.77	88.52	**98.88**	57.38	0.9178
SENet	90.04	81.01	97.61	63.05	0.9203

The highest value of each item is shown in bold

The performance of the six physicians is shown in Table 3. Senior pediatrician 2 achieved the highest accuracy (74.00%), precision (75.00%), and specificity (88.33%). Junior pediatrician 1 achieved the highest F1 score of 71.76%. The mean scores of accuracy, precision, specificity, and the F1 score of all six physicians were 63.50%, 59.91%, 67.78%, and 59.82%, respectively. All CNN models outperformed the physicians in terms of accuracy, precision, specificity, and the F1 score.

**Table 3:** Performance of each physician

	Accuracy %	Precision %	Specificity %	F1%
JP 1	60.00	75.00	88.33	**71.76**
JP 2	57.00	47.17	53.33	53.76
JP 3	59.00	48.93	60.00	52.86
SP 1	60.00	50.00	63.33	52.38
SP 2	**74.00**	**75.00**	**88.33**	61.76
SP 3	71.00	63.41	58.33	66.43
Average	63.50	59.91	67.78	59.82

JP: Junior pediatrician. SP: Senior pediatrician

The highest value of each item is shown in bold

## Discussion

Noonan syndrome is a multi-system genetic disorder that presents with development delays, congenital heart disease, renal anomalies, and a distinctive facial appearance. The facial features include a high forehead, hypertelorism, ptosis, inner epicanthal folds, down-slanting palpebral fissures, a round nasal tip, and posteriorly rotated ears. This characteristic facial morphology is an important clue for identifying NS. However, these facial characteristics are most prominent in infancy, and become less apparent with age in many people with NS. Their facial features may range from subtle to typical.

Facial recognition technology has been applied for the identification of GSs. In 2003, Loos et al. were the first to use facial recognition technology to classify five genetic syndromes. They [2] used the Gabor wavelet(GW) transform, a traditional machine learning-based method, to pre-process 55 photographs of patients of mucopolysaccharidosis type III, Cornelia de Lange syndrome, fragile X syndrome, Prader–Willi syndrome, and Williams–Beuren syndrome. A comparison of the feature vectors of 32 facial nodes led to an accuracy of classification of 42/55 (76%). Since then, a number of traditional machine learning-based methods have been developed for identifying GSs. In 2013, Zhao et al. [29] collected 100 frontal facial photographs of 50 patients with GSs as well as those of 50 healthy children to construct models of facial recognition for identifying DS. They constructed four traditional machine learning models based on the support vector machine (SVM) with the radial basis function (RBF) kernel, linear SVM, k-nearest neighbor (k-NN), and random forest (RF). The SVM with the RBF kernel achieved the best performance, with an accuracy of 94.6% and a precision of 93.3%. These results show that facial recognition technology can be used to accurately identify DS. In 2017, Kruszka et al. [10] developed a model of facial recognition to identify NS based on the SVM. They used 161 images of 161 subjects with NS from 20 countries. The facial analysis technology was able to identify NS patients in all population groups with a sensitivity and specificity of 88% and 89%, respectively. In another study, Porras et al. trained an SVM model to distinguish between patients with NS and those with Williams–Beuren syndrome, and obtained an accuracy of 85.68%. The models of facial recognition used in the above studies were all trained by using traditional machine learning-based methods. These methods require a long time for computations as well as manual feature extraction, which is laborious. In addition, the extracted features often lack a high-level representation of the face, which results in the loss of valuable information and leads to inaccurate detection [30].

With improvements in data storage and computational power, CNN has emerged as the most important method of facial recognition. The CNN is an automatic machine learning technique that does not require manually labeled images. It has exhibited impressive performance in many tasks of image classification. Porras et al. [31] developed a CNN-based model of genetic screening that used facial images of 1,400 children with 128 kinds of GSs, in addition to 1,400 matched controls. The dataset contained only one facial image from each participant. The images were obtained from three publicly available databases and the archives of the Children’s National Hospital (Washington, DC, USA). This CNN-based model achieved an accuracy of 88% and a specificity of 86% in terms of GS detection. These results show that the CNN-based model of recognition can be used to screen GS patients. DeepGestalt, a CNN-based algorithm, was introduced for identifying GS based on facial recognition in 2014. It has been incorporated into a smartphone app called Face2Gene (http://www.face2gene.com/, FDNA Inc, Boston MA USA) [32–34]. In 2023, Luis et al. [1] used Face2Gene to identify NS in a sample of Colombian subjects, and obtained a top-1 accuracy of 66.7% and a top-5 accuracy of 77.8%. In 2021, our team [35] developed a CNN model of facial recognition based on the ResNet architecture and the ArcFace loss function for identifying NS patients. The model achieved an accuracy of 92% in distinguishing between NS patients and healthy subjects. However, it recorded an accuracy of only 81% when it was used to distinguish between NS cases and other GSs. To meet the requirements of clinical practice, the performance of these models for NS identification still needs to be improved.

In the present study, we developed seven models of facial recognition for identifying NS based on VGGNet, ResNet50, MobileNet-V2, and SENet. These CNN-based classification architectures have been widely used for image recognition in recent years, and are characterized by small kernels, deep network structures, and few parameters. VGGNet uses small convolution kernels of size 3 × 3 to construct the network. The use of the residual block allows ResNet to solve the vanishing gradient problem. MobileNet is lightweight and suitable for low-power devices without GPUs. The SE block enables the network to automatically recalibrate the feature maps by selectively emphasizing informative channels. In the context of our study, these models can help physicians distinguish between NS patients and those suffering from other GSs as well as healthy children. Each CNN model outperformed the six pediatricians who were recruited for this study. In addition, the models based on the VGG network series achieved good performance. Similar results have been obtained in other studies on few-shot learning in the domain of medical image-based recognition. Krushi Patel et al. [36] constructed five models for classifying colorectal polyps based on different CNN architectures (VGG, ResNet, DenseNet, SENet, and MnasNet). The training set included 119 endoscopy videos of patients suffering from colorectal polyps. The VGG network achieved the best performance with an accuracy of 79.78%. In 2021, Liu et al. [37] developed five models of facial recognition for William syndrome patients by using the VGG16, VGG19, ResNet18, ResNet34, and MobileNet-V2 architectures respectively. The VGG19 model achieved the best performance, followed by the VGG16 model. The authors presumed that the VGG network series might be more suitable than the other networks for recognition tasks involving a limited number of samples [38].

BN is a technique for training deep neural networks that standardizes the inputs to a layer for each mini-batch. It aims to reduce the internal covariate shift to accelerate the training process [23]. BN can provide three major benefits. Firstly, it increases the training speed by normalizing inputs of each layer to have zero mean and unit variance. Secondly, BN acts as a regularizer and allow the model to converge with a high learning rate. Thirdly, BN is able to prevent over-fitting, so it can replace Dropout and Local Response Normalization to simplify the network [39]. BN also has a beneficial effect on gradient flow through the network by reducing the dependence of the gradients on the scale of the parameters and their initial values. In this study, the VGG19-BN model achieved the best overall performance, with the highest accuracy, precision, and specificity. The VGG16-BN model achieved the highest score (0.9787) in terms of the AUC, followed by the VGG19-BN model (0.9415). The addition of BN can thus improve the performance of the VGG model. One study [38] on the automatic image classification showed that BN can accelerate the convergence of the model, improve its precision, and reduce anticipation loss.

### Limitations

This study has four main limitations: (1) A reliable diagnostic model typically relies on a sufficiently large dataset. As NS is a rare genetic disease, a limited number of facial images were collected for this study. (2) Only a portion of the healthy children considered here were genetically tested, while the other healthy children were evaluated by two pediatric geneticists to exclude the presence of GSs. Thus, some of them might have been undiagnosed patients of GSs. However, this probability is extremely low, as none of healthy children manifested any symptoms of GSs. (3) A suitable training set for models of facial recognition should include information from a multi-racial population. Only one patient in our center was from the Zhuang ethnic group, while all the other GSs patients were ethnic Han. (4) The facial features of NS are most prominent in infancy and become less apparent with age. Owing to the limited sample size, we did not stratify the data according to the age of the patients when developing models of facial recognition. We plan to collect more data on NS patients of different ages to optimize our models in future work.

## Conclusion

The results of our study demonstrate that the computer-assisted model of facial recognition can improve the diagnosis of NS. The models of facial recognition based on VGG-19 BN and VGG-16 BN can thus play an important role in the diagnosis of NS in clinical practice.

### Electronic supplementary material

Below is the link to the electronic supplementary material.

Supplementary Material 1

## Data Availability

All data used during the study are available from the corresponding authors upon reasonable request.

## Abbreviations

NS: Noonan syndrome
GSs: genetic syndromes
MTCNN: multi-task convolutional neural network
SENet: Squeeze-and-Excitation Network
CNN: convolutional neural network
DS: Down syndrome, TS: Turner syndrome, MAPK: mitogen-activated protein kinases, GMDB: GestaltMatcher database
P-Net: proposal network
R-Net: refinement network
O-Net: output network
BN: Batch normalization
TP: true positive
TN: true negative
FP: false positive
FN: false negative
ROC: receiver operating characteristic
AUC: area under the curve
GW: Gabor wavelet
SVM: support vector machine
RBF: radial basis function
k-NN: k-nearest neighbor
RF: random forest
