# Supplementary material for: Computer-based facial recognition as an assisting diagnostic tool to identify children with Noonan syndrome
Source: BMC Pediatr. 2024 May 24;24:361. doi: 10.1186/s12887-024-04827-7 (PMC11118109; doi:10.1186/s12887-024-04827-7)
Supplement: Supplementary file 1 — Supplementary Material 1 [file 12887_2024_4827_MOESM1_ESM.docx]

Supplemental File 1

| Period | Age  （years） | Patients collected from hospital(n=83) | Patients collected from literature and GMDB(n=155) |
| --- | --- | --- | --- |
| Infant period | ＜1 | 25 | 17 |
| Toddlers’ age | 1-2 | 24 | 61 |
| Preschool age | 3-5 | 17 | 29 |
| School age | 6-11 | 7 | 42 |
| Adolescence | 12-17 | 5 | 6 |
